# Delta9-THC determination by the EU official method: evaluation of measurement uncertainty and compliance assessment of hemp samples

**DOI:** 10.1007/s00216-021-03283-x

**Published:** 2021-03-23

**Authors:** Salvatore Sgrò, Benedicta Lavezzi, Cristian Caprari, Marco Polito, Marcello D’Elia, Giampietro Lago, Giada Furlan, Stefano Girotti, Elida Nora Ferri

**Affiliations:** 1 Chemical Laboratory of Bologna, Anti-Fraud and Controls Office – Laboratories Section, DT VII, Italian Customs and Monopolies Agency, V. le P. Pietramellara 1/2, 40121 Bologna, Italy; 2 Department of Pharmacy and Biotechnology, University of Bologna, Via San Donato 15, 40127 Bologna, Italy; 3 Scientific Police Center for Emilia-Romagna Region, Via Volto Santo 3, 40123 Bologna, Italy; 4 Carabinieri Scientific Investigation Department (RIS) of Parma, Parco Ducale 3, 43120 Parma, Italy

**Keywords:** Measurement uncertainty, Hemp, THC, GC-FID, *Cannabis* light, Compliance assessment

## Abstract

Hemp cultivation is living a period of renewed interest worldwide after long years of opposition and abandonment. The European Union (EU) allows and subsidizes the growing of fiber and oilseed cultivars of *Cannabis sativa* L. with respect to the THC content limit of 0.2%. The EU method for the quantitative determination of Δ9-tetrahydrocannabinol (THC) content in hemp varieties provides to apply a tolerance of 0.03 g of THC per 100 g of sample concerning compliance assessment to that limit. However, the method does not report any precision data, especially useful as a function of THC content to evaluate measurement uncertainty and therefore to establish the conformity of hemp at different THC legal limits. Measurement uncertainty of the method by both bottom-up and top-down approach, besides repeatability and reproducibility, was investigated and estimated in the THC concentration range 0.2–1.0%, which includes the different legal limits set out for hemp around the world. We proposed Decision Rules for conformity of hemp showing that a non-compliant declaration beyond reasonable doubt should be stated when the THC content, as a mean result on a duplicate analysis, exceeds the limit by at least 11–15%, depending on THC limit. We highlighted other issues concerning practical aspects of hemp analysis, from sampling to evaluation of results, as well as the need to carry out collaborative studies on the EU method.

**Supplementary Information:**

The online version contains supplementary material available at 10.1007/s00216-021-03283-x.

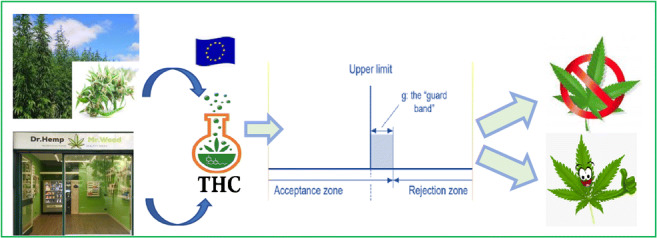

## Introduction

*Cannabis sativa* L. is the world’s most recognizable, notorious, and controversial plant known since the ancient times for its medicinal and textile uses, an emblematic example of a multi-purpose crop [1, 2]. It is also by far the most widely cultivated, trafficked, and abused illicit drug [3].

**Fig. 1:**
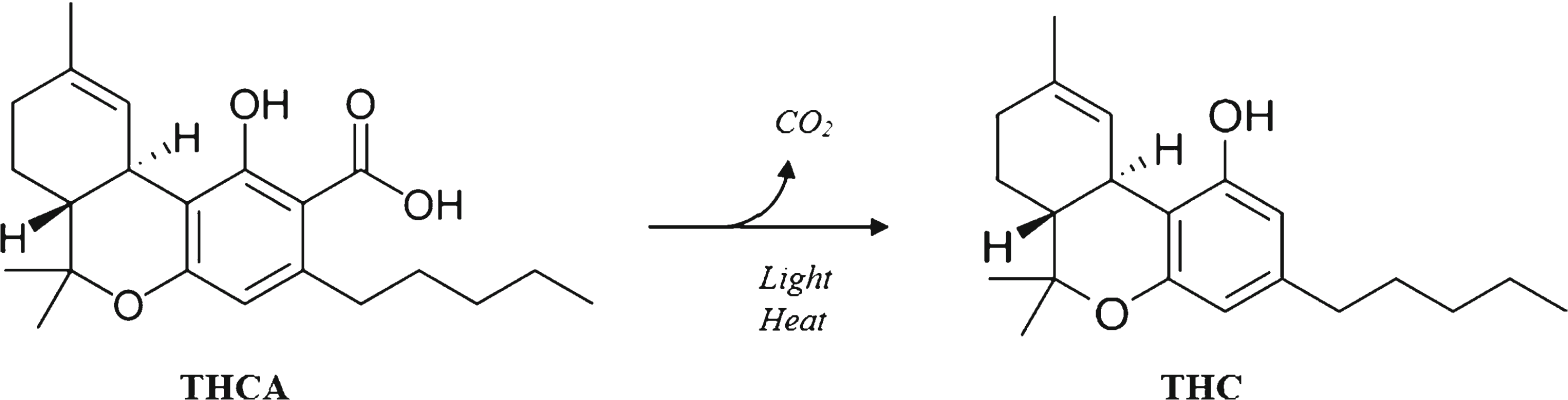
The decarboxylation reaction of THCA to THC

The name “hemp” or “industrial hemp” designates fiber and oilseed cultivars of *C. sativa* with very limited content of Delta-9-tetrahydrocannabinol (Δ9-THC, or simply THC). Conversely, “marijuana” is the name used for the drug kind of plant, containing a high level of THC. THC and CBD (cannabidiol) are the plant phytocannabinoids of most importance. THC is the principal intoxicant and psychotropic constituent, while CBD, devoid of psychotropic effects and known to possess several pharmacological properties, is instead the principal cannabinoid of hemp [4]. These compounds, as other cannabinoids, exist in the fresh plant mostly in the form of carboxylic acids, THCA and CBDA, possessing several pharmacological properties but no psychotropic activity [5]. These acids undergo decarboxylation into their neutral counterparts under the influence of light, time (such as prolonged storage), alkaline conditions, or high temperature (smoked or cooked marijuana) following the reaction shown in Fig. 1.

Marijuana has been considered a leading drug of abuse and has been seriously criminalized, with enormous law enforcement costs and social upheaval [1]. It is currently included in Schedule I of the United Nation (UN) Single Convention on Narcotics Drugs (1961) [6] and only recently it was removed from Schedule IV, the most restrictive [7]. In the last decade, decriminalization of *Cannabis* for industrial and medicinal uses and even recreational marijuana has occurred, or it is occurring in many jurisdictions as the result of sociological, philosophical, political, and legal developments [8]. A limit of 0.3% of THC content (on a dry inflorescences weight basis) was established by Small et al. (1976) [9] and adopted in many countries as a criterion to distinguish cultivars that can be legally cultivated under license from those considered to have a too high drug potential [1]. Some jurisdictions have increased this limit for legal cultivars up to 1.0% [1, 10, 11].

In Europe, the THC limit value for industrial hemp was first set at 0.5% in 1984, then trimmed to 0.3% in 1987, and further lowered to 0.2% in 1999 to prevent the cultivation of illicit drug–type *Cannabis* in hemp fields [12]. The EU subsidies to hemp cultivation are granted upon the use of certified seeds from the varieties listed in the “Common Catalogue of Varieties of Agricultural Plant Species” [13], provided that THC content does not exceed 0.2% [14]. Hemp imported in the EU must meet the same limit [15].

In Italy the Law n. 242/2016 [16], laying down rules for support and promotion of the hemp cultivation, stated that farmers were not liable to the Italian narcotics law [17] when hemp THC content, higher than 0.2%, did not exceed the 0.6%. This led to the misinterpretation of the Law as a general liberalization of *Cannabis* derivatives having a THC content below 0.6%, the so-called *Cannabis light*. The consequent booming demand of *C. light* and the specialized stores opening (+200% from 2016) [18] quickly became an economic and social phenomenon, giving rise to many jurisdictional controversies and to several seizures. A note of the Italian Ministry of Interior stated, in 2018, that *Cannabis* inflorescences, plants, concentrates, essences, and resins on the market were considered narcotics when the THC content was higher than 0.5%, according to a judgment of the Italian Supreme Court of Cassation (1989) based on forensic toxicology studies, scientific literature, and court judgments [19]. In 2019, the Italian Supreme Court (plenary session) stated that the marketing of leaves, inflorescences, oil, and resin of *Cannabis sativa L.* was out of the scope of the Law n.242/2016 and then it is an offense under the Italian drug control law, “except if the products are in practice devoid of narcotic effects.”

Actually, the current legal framework in this field needs to be harmonized and better defined to avoid ambiguous interpretations and contradictory judgments. Anyway, Authorities and law enforcement agencies worldwide have in charge to analyze *Cannabis* derivatives verifying its compliance with different THC legal limits. As required by ISO/IEC 17025:2017, the applied decision rules must be clearly defined when reporting about compliance, and the knowledge of uncertainty associated with the measurements is essential to this purpose. Without this information, there is a risk of misinterpretation of results, incorrect prosecution in law, adverse health, or social consequences [20].

A widely adopted standard approach to uncertainty evaluation is the Guide to the Expression of Measurement Uncertainty (GUM) [21], using a bottom-up approach. On the other hand, it is possible to carry out collaborative studies on standard test methods, and measurement uncertainty evaluation is achieved using precision and trueness estimates (top-down approach) [22].

Literature reports several analytical methods for determining phytocannabinoids in *Cannabis* plants and derivatives, most of which based on gas chromatography coupled to flame ionization (GC-FID) or mass spectrometry (GC-MS) detector, or on high-performance liquid chromatography coupled to ultraviolet (HPLC-UV) or mass spectrometry (HPLC-MS) detector [5, 23–30]. Each method has inherent limitations and many are the pitfalls encountered in *Cannabis* analysis, from sampling to sample preparation and from cannabinoids extraction to instrumental analysis [31]. In particular, these issues may be highly critical in the analysis of samples with low content of THC.

The official method established by the European Commission employs a GC-FID analysis [32]. However, this method does not report any precision data, useful especially as a function of THC content to measurement uncertainty evaluation and therefore to establish the conformity of hemp at different THC legal limits. It reports applying a tolerance of 0.03 g of THC per 100 g of sample for compliance to the limit of 0.2% THC content.

Therefore, we considered it necessary to investigate and estimate the precision data of the EU method at different THC concentration levels, representing the THC legal limits set out for hemp around the world: 0.2%, 0.3%, 0.5%, 0.6%, and 1.0%. We verified the specificity of the method by GC-MS analysis and its trueness by comparing its data with those obtained by a GC-FID method validated and accredited at the Italian Customs and Monopolies Agency Laboratories (MAD) [33]. We evaluated the measurement uncertainty for each one of the abovementioned legal limits by both bottom-up and top-down approaches and decision rules for compliance assessment of hemp were proposed.

## Materials and method

### Reagents and solvents

Δ^9^-tetrahydrocannabinol (Δ^9^-THC, 1.0 mg/ml in methanol) and Δ^9^-tetrahydrocannabinoid acid A (THCA, 1.0 mg/ml in acetonitrile) were supplied by Cerilliant (Round Rock, Texas), squalane (analytical Internal Standard, IS, > 99%) was purchased from Merck (Darmstadt, Germany), and n-hexane (99%) was purchased from Carlo Erba (Milano, Italy). The dried hemp inflorescence samples were seized by Authorities or delivered in the analysis by growers and retailers.

### Sample preparation

First, we verified the moisture content in the samples. Shortly, about 3 g of each sample was accurately weighed and further dried for 4 h at 103 °C in an oven [34], weighted again, and then discarded.

The samples, according to the EU method [32], were ground to a semi-fine powder (passing through a 1-mm mesh sieve), after removing stems and seeds over 2 mm in size. We placed 100 mg of the powdered sample in a centrifuge tube adding 5 ml of extraction solution containing the internal standard (IS) (35 mg of squalane per 100 ml hexane). The sample was placed in an ultrasound bath for 20 min, then centrifuged for 5 min at 1390 g. The supernatant, containing the THC, was removed and placed in a vial for GC analysis.

In particular, we selected, or prepared by properly mixing the available real ones, five samples at different concentration levels, ranging from about 0.1 to 1.0% m/m of THC.

### Calibration curve

The calibration standard solutions were prepared by drying a proper volume of the THC standard solution in methanol under N_2_ flow and diluting it by the IS extraction solution. The calibration curve included 5 calibration levels, 0.02, 0.04, 0.12, 0.25, and 0.5 mg/ml of THC, corresponding to a THC amount in samples ranging from 0.1 to 2.5% m/m. In particular, the 0.04 and 0.5 mg/ml standard solutions were those required by the EU method. We injected 3 times each standard solution and plotted the THC and IS peaks area ratio vs their concentration ratio.

The calibration curve equation was calculated by the least squares regression and linearity evaluated by the coefficient of determination and normalized residuals. The limit of detection (LOD) and the limit of quantification (LOQ) were estimated on a 3:1 and a 10:1 signal-to-noise ratio, respectively.

### GC-FID analysis

The GC-FID analyses according to the EU method were performed on a Shimadzu GC-2010 Plus SSL/FID with autosampler (Shimadzu Corporation, Kyoto, Japan), equipped with a Restek Rxi-5-ms fused silica capillary column, 30 m × 0.25 mm i.d., and 0.25-μm film thickness (cross-linked 5% diphenyl-95% dimethylpolysiloxane). The carrier gas was helium, at a flow rate of 1 ml/min. The injection volume was 1 μl with a 1:40 split ratio. The oven temperature was set to 260 °C × 10 min, then to 300 °C (20 °C/min) × 2 min. The injector and FID temperature was set at 300 °C, the latter fed by a flow of H_2_ (40 ml/min), air (400 ml/min), and N_2_ (30 ml/min) as make-up gas. Each single run lasted 14 min.

The THC amount (*y*) was calculated by the following formula:

1 $$ THC\left(\%\right)=\frac{A_{\mathrm{THC}}/{A}_{\mathrm{IS}}-a}{b}\ast \frac{C_{\mathrm{IS}}\kern0.75em {V}_{\mathrm{s}\mathrm{ol}}}{w_{\mathrm{s}}}\ 100 $$

where *a* and *b* are respectively the intercept and the slope of the linear regression equation, *A* is the chromatographic peak area, *C* is the concentration in mg/ml, *V*_sol_ is the extraction solution volume in ml, and *w*_s_ is the sample weight in mg.

The analyses according to the MAD method [33] were performed on the same GC-FID instrument. This method differs from the EU one in the extraction solution (IS and solvent) and in the GC temperatures.

### GC-MS analysis

The GC-MS analyses to identify the extracted compounds and to evaluate the method specificity were performed on a Thermo Focus GC/DSQ II with autosampler (Thermo Fisher Scientific, MA, USA). The column and the GC conditions were the same reported above for the EU GC-FID assay. The MS detection was performed by electron ionization (EI) at 70 eV, operating in full scan acquisition mode in the *m/z* range 40–450. The interface and ion source temperatures were set at 270 and 250 °C, respectively. The reference standard solutions were run under the same conditions and mass spectra matches were carried out using the National Institute of Standards and Technology (NIST) mass spectra database (version 2.2, 2014).

### Statistical analysis

The precision, trueness, and uncertainty of the THC content measurement were determined by following international guidelines [35–38] and by applying standard statistical treatments to experimental data. The variability (repeatability) associated to the THC content was studied by the Shapiro-Wilk test to verify the normal distribution of data and by Dixon, Grubbs, and Huber test to remove the outlier data. We applied the *t*-test, the *F*-test, and the Hartley test to study the means and the variances, respectively.

The Horwitz ratio (HorRat), a performance parameter reflecting the acceptability of a chemical method of analysis with respect to precision, was applied to evaluate the within-laboratory variability [39].

We determined the measurement uncertainty, which characterizes the dispersion of the values reasonably attributed to the analytes, by both the bottom-up approach [21] and the top-down one [22].

## Results and discussion

### Specificity of the method

**Fig. 2:**
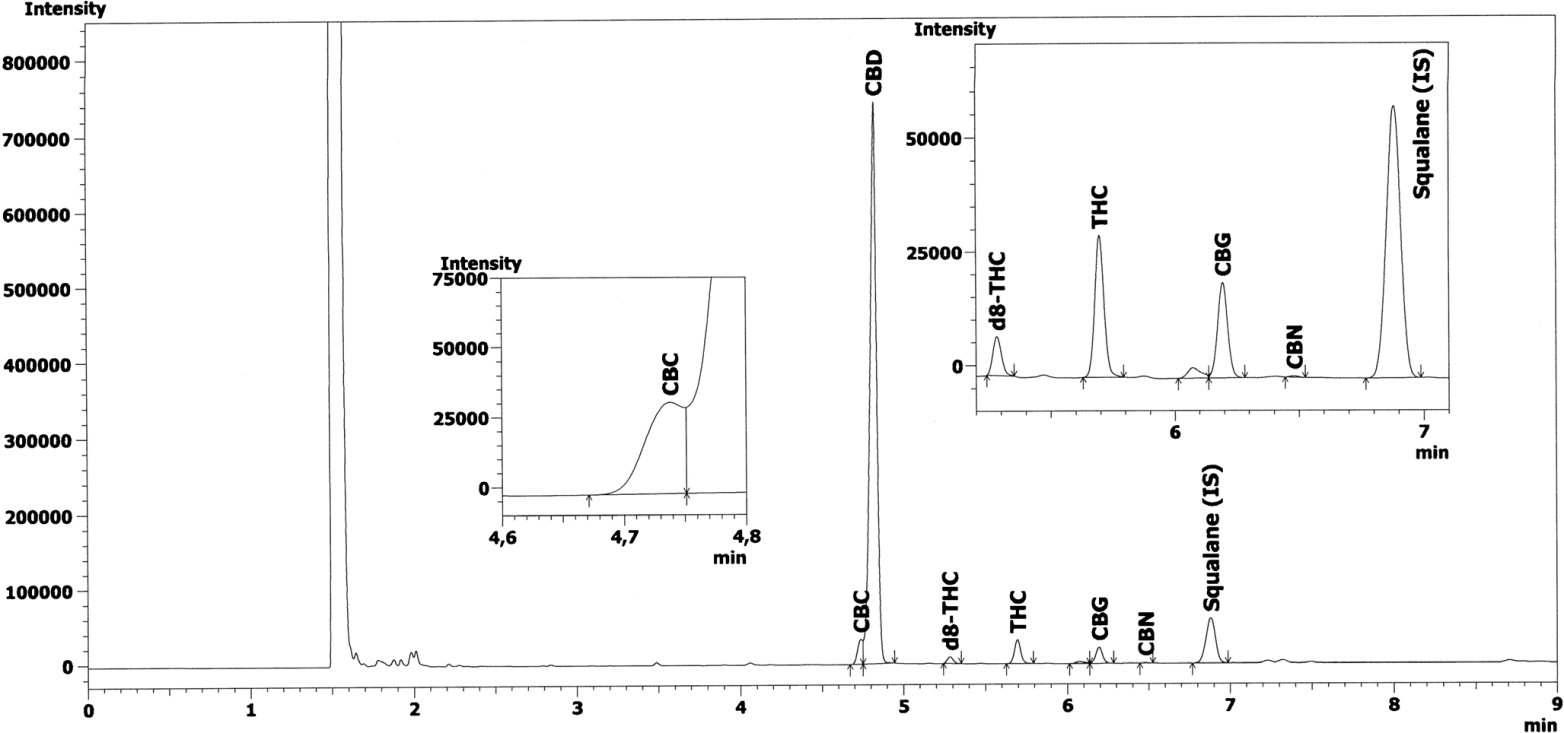
GC-FID chromatogram of the extract from a hemp sample with low content of THC

**Fig. 3:**
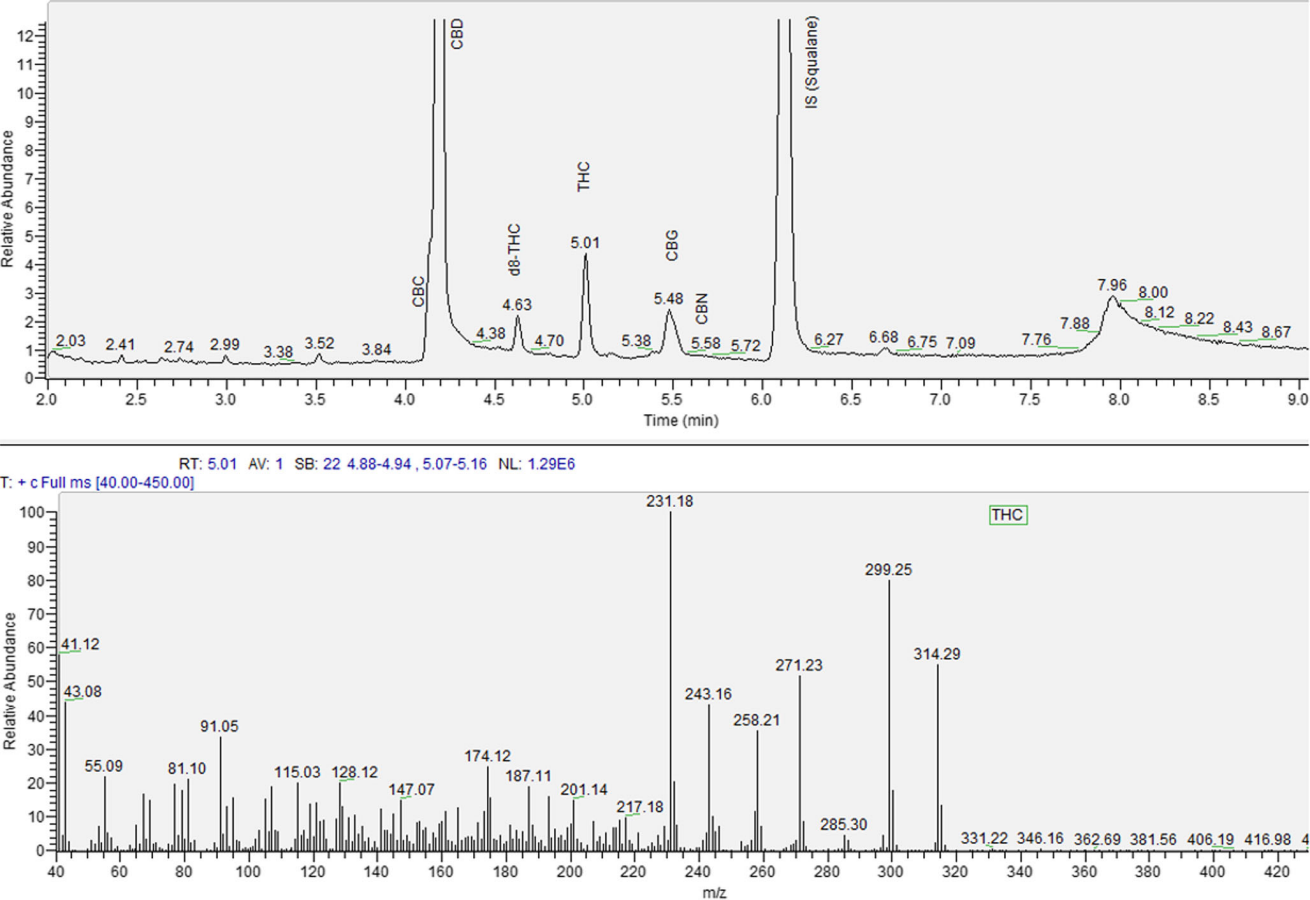
GC-MS chromatogram and mass spectrum of the extract from a hemp sample with low content of THC

Some sample extracts underwent analysis by both GC-FID and GC-MS assays to ascertain the specificity of the EU method. The same profiles and similar peak retention times resulted from the two techniques. For the THC and the IS peaks, a resolution higher than 1.3 occurred in all samples. We obtained the identification of the different cannabinoids by comparing our retention time of peaks and MS fragmentation patterns with those of the reference standards (Figs. 2 and 3). The method, therefore, resulted specific.

### Calibration curve

The calibration curve equation for the GC analyses resulted *y* = 0.8216*x* − 0.0155, with *R*^2^ = 0.9995 and normalized residuals <2. The limit of detection (LOD) and quantification (LOQ) resulted equal to 0.003 mg/ml and 0.010 mg/ml, respectively. These values correspond to a THC content of 0.015% and 0.050% m/m, less than one tenth and equal to one fourth of the legal limit of 0.2%.

### Precision

The five selected or prepared samples were analyzed by performing eleven independent replications, in order to assess the variability (repeatability) associated to the THC level, which was in the range 0.10–1.11% m/m of THC. The moisture content in all samples was in the range 8–13%, as required by the EU method.

The results of data statistical treatment are reported in Table 1. For each concentration level, we calculated:

- The mean measured value of THC (%),
- The repeatability (within-laboratory) standard deviation (s_r_),
- The relative standard deviation (RSD_r_,%),
- The predicted reproducibility (among-laboratory) standard deviation calculated by the Horwitz equation (σ_H_(%m/m) = 2*C*^0.8495^), where *C* is the THC content as mass fraction m/m),
- The predicted relative standard deviation (PRSD_R_ = σ_H_/THC,%),
- The Horwitz ratio (HorRat_r_ = RSD_r_/ PRSD_R_).

**Table 1:** THC determination (GC-FID) repeatability data and Horwitz ratio (HorRat_r_) values

THC (%)	*n*	*s*_r_	RSD_r_ (%)	*σ*_H_	PRSD_R_ (%)	HorRat_r_
0.10	11	0.00619	6.2	0.00566	5.7	1.1
0.19	11	0.00902	4.7	0.00976	5.1	0.9
0.31	11	0.01206	3.9	0.01479	4.8	0.8
0.58	9	0.02001	3.5	0.02518	4.3	0.8
1.11	11	0.04160	3.7	0.04371	3.9	1.0

The Hartley test on variances allowed establishing that variability of the analysis was dependent from THC level, being:

$$ {F}_{\mathrm{calc}}\left({s^2}_{\mathrm{r}\ \max }/{s^2}_{\mathrm{r}\ \min}\right)>{F}_{\max \left(1-\upalpha; \mathrm{p};\upupsilon \right)}, $$

where *α* = 0.05 (significance level); *p* = 5 (number of levels); *υ* = 10 (degrees of freedom).

In particular, the standard deviation *s*_r_ showed a linear dependence from the THC % m/m in the investigated range, which equation was *y* = 0.035*x* + 0.0018 with an *R*^2^ = 0.9918.

This result confirmed that our choice to analyze just only the five samples corresponding to the legal limits set around the world led to statistically significant results.

The original Horwitz ratio, HorRat_R_ (RSD_R_/PRSD_R_, where RSD_R_ is the relative reproducibility standard deviation obtained by an inter-laboratory study), has empirical acceptable values in the range 0.5–2.0 [39]. To the best of our knowledge, collaborative trials on the UE method were not performed yet, nor were available precision data (*s*_R_) for the analysis of THC low-level contents.

Anyway, the Horwitz ratio can be also applied to within-laboratory precision (HorRat_r_), although with less reliability. Since the within-laboratory variability (*s*_r_) is typically one half to two thirds of the among-laboratory variability (*s*_R_) [39], the HorRat_r_ acceptable range shall be 1/4–4/3. As shown in Table 1, at all analyzed THC levels, the required condition was satisfied.

### Trueness

Certified reference materials (CRM) for hemp were not available and we evaluated the trueness of data by two assays.

We employed a THC standard solution at 0.1 mg/ml, an intermediate level of calibration, corresponding to the 0.5% amount in a sample. We injected this standard sample in triplicate and the recovery values resulted in the range 99.8–100.3%.

In parallel, we compared the results obtained on six replicates of a randomly selected real sample, analyzed by both the EU method and the MAD one. We applied the *t*-test and the *F*-test to the mean and variance values. It was noteworthy that the two GC-FID methods were perfectly comparable. In fact, the mean value was 0.28% for both methods and the standard deviation resulted 0.0099% for the EU method and 0.0072% for the MAD one.

Moreover, we decided to evaluate the THCA conversion and recovery values in our GC system. Indeed, THC is mostly present as a carboxylated form in fresh *Cannabis* plants as well as in samples dried at low temperature and not too old. The EU method, in fact, provides to dry fresh hemp samples below 70 °C, since at higher temperature (85–100 °C) the THC decomposition may occur. THCA decarboxylation reaction starts around 60–90 °C [40–44] and conversion to THC was never perfectly complete without loss or degradation of the starting material [45, 46].

As already reported by Dussy et al. [43], the THCA decarboxylation reaction occurring in a gas chromatograph does not have a fixed rate as it strictly depends on the liner geometry and injector port temperature. It was referred a thermal conversion of THCA of only 70% at the maximum.

Therefore, we employed a THCA standard solution (STDS) at three different concentrations, each one injected three times and results were quantified as THC. We reported the results in Table 2.

**Table 2:** THCA recovery values evaluated from the comparison between the expected THC values (THCA*0.877, reported also as % in sample) and the measured ones

THCA (STDS)	Expected THC		Measured THC	Recovery	
mg/ml	(mg/ml)	(% m/m)	mg/ml	(%)	
0.046	0.040	0.20	0.041	101.5	
0.140	0.123	0.61	0.117	95.0	
0.250	0.219	1.10	0.214	97.6	
				Mean	98.0

The THCA mean recovery value denoted a satisfactory performance of our GC system relating to the THCA conversion rate throughout the concentration range of our interest.

In any case, a correction of the results by THCA recovery is neither advisable nor is it effectively possible, since the actual THCA content in *Cannabis* products cannot be determined by a GC analysis without any prior derivatization. Nevertheless, THCA recovery and conversion rate determination are recommended to verify and ensure the own GC is suitable to perform the analysis [23].

In general, it is possible to affirm that *Cannabis* analysis by GC may cause a variable underestimation of total THC content.

### Measurement uncertainty

#### The bottom-up approach

This methodology evaluates the measurement uncertainty focused on individual input quantities. In general, this approach may underestimate the measurement uncertainty, mainly because it can be difficult to identify and include all possible contributions.

Shortly, the combined standard uncertainty *u*_c_(*y*) associated to a result (***y***) is determined from the estimated standard deviation associated with each input ***x***_**i**_**,** the standard uncertainty *u* (*x*_i_). Some of these inputs are evaluated from the statistical distribution of the results and are characterized by experimental standard deviations (type A evaluation). Other inputs, also characterized by standard deviations, are evaluated from assumed probability distributions based on experience or on other information (type B evaluation).

The relative combined standard uncertainty,${\dot{\ u}}_c(y)$, considering the Eq. (1), was calculated as:

2 $$ {\dot{u}}_c(y)=\sqrt{{\dot{u}}_{rep}^2+{\dot{u}}_{reg}^2+{\dot{u}}_{CRM(THC)}^2+{\dot{u}}_{CRM(IS)}^2+{\dot{u}}_{w_s}^2+{\dot{u}}_{V_{sol}}^2} $$

by combining the contributions of repeatability (*u*_rep_), a type A evaluation, with those of regression curve (*u*_reg_), THC certified reference material (*u*_CRM(THC)_), IS certified reference material (*u*_CRM(IS)_), sample weight (*u*_ws_), and extraction solution volume (*u*_Vsol_), representing type B evaluations.

In case the moisture content does not fall within the range provided by the method and a correction is therefore necessary, the related contribution should be included in the uncertainty budget as well.

The expressions employed to calculate each contribution value are provided in the Supplementary Information (ESM).

It is appropriate to underline that we used a modified version of the well-known formula for *u*_reg_ calculation, specifically omitting the first term “1/*m*” (where *m* is the number of replicates) provided under the radical. Indeed, it is necessary to avoid double counting the precision contribution, already accounted in the uncertainty budget with the repeatability term ${\dot{u}}_{rep}$ that incorporates all the individual sources of variability, including that relating to calibration, as well explained by Kadis [47].

For each legal limit in the range 0.2–1.0% of THC, the combined standard uncertainty, *u*_c_(*y*) = *y*${\dot{u}}_c(y)$, and the expanded uncertainty, *U*(*y*) = *k u*_c_ (*y*), were calculated both for one (*m* = 1) and two (*m* = 2) determinations per test sample.

Expanded uncertainty was calculated using a coverage factor *k* = 2, as the effective degrees of freedom resulted *ν*_eff_ > 10, providing a level of confidence of approximately 95%.

In Table 3, we reported the values of the abovementioned inputs and the combined relative standard uncertainties, whereas in Table 4, the standard, expanded, and relative expanded uncertainty values.

**Table 3:**
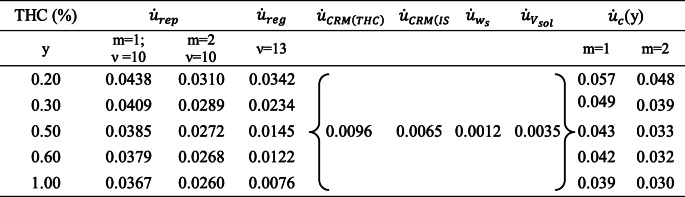
Values of the components of bottom-up uncertainty and the combined relative standard uncertainties (*ν* = the degrees of freedom; *m* = number of replicates)

**Table 4:** Evaluation of the combined, expanded, and relative expanded uncertainty by the bottom-up approach (coverage factor *k* = 2)

THC (%)	*u*_*c*_(y)				*k*	*U*(*y*)		$\dot{U}$(*y*) %	
*y*	*m* = 1	*ν*	*m* = 2	*ν*		*m* = 1	*m* = 2	*m* = 1	*m* = 2
0.20	0.011	22	0.010	26	2	0.02	0.02	11.4	9.5
0.30	0.015	19	0.012	25		0.03	0.02	9.7	7.8
0.50	0.022	15	0.017	21		0.04	0.03	8.6	6.6
0.60	0.025	14	0.019	19		0.05	0.04	8.3	6.4
1.00	0.039	13	0.030	17		0.08	0.06	7.9	5.9

The ${\dot{u}}_{rep}$ and the ${\dot{u}}_{reg}$ values depend on THC amount and were extrapolated by the respective correlation functions.

The correlation values and function of ${\dot{u}}_{reg}$ with the THC content (both mg/ml and %) are reported in Table S1 and Fig. S1 in the ESM.

The calibration uncertainty *u*_cal_, given by the appropriate sum of *u*_reg_, *u*_CRM(THC)_, and *u*_CRM(IS)_, represented about 40% (60% for *m* = 2) of the global uncertainty at the 0.2% THC. It decreased to 10% (20% for *m* = 2) for the 1.0% limit. A graph (Fig. S2) showing the contribution of each input to the combined standard uncertainty at each legal limit was included in the ESM.

#### The top-down approach

The measurement uncertainty can be evaluated also by using the repeatability, reproducibility, and trueness data obtained by collaborative studies conducted in accordance with ISO 5725-2 [22, 48], following the principle that reproducibility standard deviation obtained on a collaborative study is a valid basis for measurement uncertainty evaluation.

According to ISO 21748/2017 [22], we calculated the standard uncertainty by the following equation:

3 $$ u(y)=\sqrt{s_R^2-{s}_r^2\left(1-\frac{1}{m}\right)} $$

Hence, the expanded uncertainty is calculated as *U*(*y*) = *ku*(*y*), where *k* = 2.

Since *s*_R_ information from collaborative study on EU method are unavailable to date, we calculated a rough estimate of *s*_R_ from *s*_r_, as:

4 $$ {s}_{\mathrm{R}}=2\ {s}_{\mathrm{r}}\kern0.5em =0.0699x+0.0035 $$

(x = % THC content), considering that the “Horwitz ratio” (*s*_R_/*s*_r_) for analytical procedures is typically close to 2.0 and does not change significantly with the concentration of the analyte [49]. This calculation of *s*_R_ was still within the acceptable range of the HorRat_R_ parameter, being less than two.

In the case of one determination per test sample (*m* = 1), as required by procedure A of the EU method, the expanded uncertainty resulted from:

5 $$ U(y)=2\ u(y)=2\ {s}_{\mathrm{R}}=4\ {s}_{\mathrm{r}}\kern0.5em =0.1398x+0.0071 $$

In the case of duplicate determinations (*m* = 2), procedure B, as it is required when the single result is above the allowed limit, the equation became:

6 $$ u(y)=\sqrt{s_R^2-\frac{1}{2}{s}_r^2}=\frac{\sqrt{14}}{2}{s}_r,\mathrm{and}\ \mathrm{the}\ \mathrm{expanded}\ \mathrm{uncertainty}:U(y)=\sqrt{14}{s}_r. $$

In Table 5, we reported the standard, expanded, and relative expanded uncertainty values obtained by the top-down approach.

**Table 5:** Top-down evaluation of the standard, expanded, and relative expanded uncertainty

THC (%)	*u*(*y*)		*k*	*U*(*y*)		$\dot{U}$(*y*) %	
*y*	*m* = 1	*m* = 2		*m* = 1	*m* = 2	*m* = 1	*m* = 2
0.20	0.018	0.016	2	0.04	0.03	17.5	16.4
0.30	0.025	0.023		0.05	0.05	16.3	15.3
0.50	0.039	0.036		0.08	0.07	15.4	14.4
0.60	0.045	0.043		0.09	0.09	15.2	14.2
1.00	0.073	0.069		0.15	0.14	14.7	13.7

The top-down approach may not by itself identify where the major errors occur and the results depend on technical competence of the laboratory concerned.

#### Comparison of the uncertainties values

The bottom-up and top-down standard uncertainties were compared for each legal limit by the *F*-test and were considered significantly different at a 95% confidence level. For both approaches, the relation between repeatability and uncertainty, *r* (= 2$\sqrt{2}$*s*_r_) < 2 *U*, was verified.

**Fig. 4:**
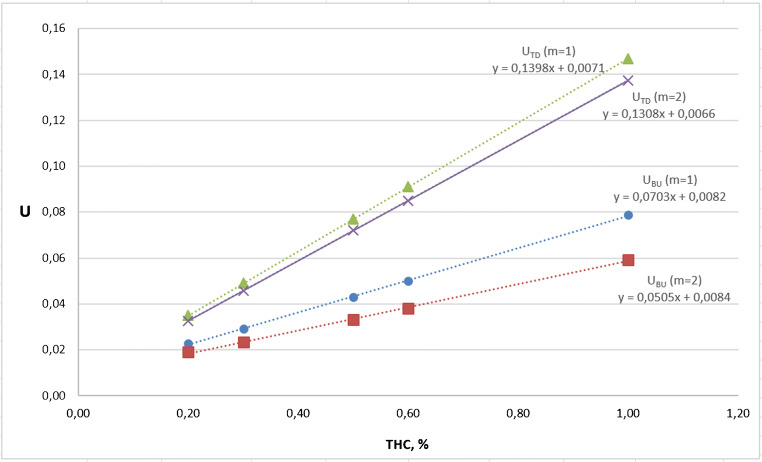
Linear correlation between top-down (TD) or bottom-up (BU) expanded uncertainties (for *m* = 1 and *m* = 2) and the THC content

We ascertained the linear correlation existing between the expanded uncertainties and the THC content, for both approaches, as shown in Fig. 4: the top-down uncertainty resulted about 1.5–2 times larger than the bottom-up one.

In Table S2 in the ESM, all the ratios of the combined standard uncertainties for *m* = 1 and *m* = 2 were reported together with the predicted standard uncertainty.

We supposed that the bottom-up approach underestimated the measurement uncertainty in hemp analysis. Indeed, the bottom-up standard uncertainty resulted quite similar to the repeatability standard deviation *s*_r_, although also comparable to the predicted reproducibility standard deviation (*σ*_H_) calculated by the Horwitz equation [39]. However, in relation to the latter aspect, it is possible to hypothesize a larger predicted variability: recent collaborative trials [49] showed a *s*_R_ significantly higher than that one provided by Horwitz, resulting much closer to the top-down standard uncertainty obtained.

Hence, inter-laboratory studies on hemp samples with low THC content will be necessary to estimate the actual precision data of the EU method. The collaborative trials (CT) will be the most suitable for this purpose. The proficiency tests (PT) do not usually prescribe specific methods and the inter-method differences will increase dispersion and uncertainty associated to the results. Statistical treatments showed that the inter-laboratory standard deviation *s*_R**’**_ under PT conditions is higher than *s*_R_ under CT ones, on average *s*_R**’**_ ≈ 1,5*s*_R_ [49, 50].

The uncertainty may result even larger considering the issue of THCA conversion variability and other sources of uncertainty, not accounted even by a collaborative study, such as sampling, removing stems and seeds (sub-sampling), and drying and grinding of samples.

We considered the top-down uncertainty estimated in this work as the minimum one to be associated to the result and the compliance assessment of hemp was based on it.

### Compliance assessment

The decision rules give a prescription for the acceptance or rejection of a product based on the measurement result, its uncertainty, and the specification limit or limits, taking into account the acceptable level of the probability of making a wrong decision [51–53].

We proposed a decision rule for non-compliance with a low probability of false rejection (high confidence of correct rejection) consistent with that one adopted by the EU method, allowing a tolerance beyond the admitted limit. Currently, a widely used decision rule implies non-compliance with an upper limit if the measured value exceeds the limit by the expanded uncertainty [51]. Anyway, the use of guard bands is preferred, as it can reduce the probability of making an incorrect conformance decision [52]. The rejection zone starts at the value of the specification limit L plus an amount g (guard band). The value of g depends upon the value of the uncertainty and the values resulting in greater than *L* + *g* have a probability of false rejection lower than the risk “*α*” [51], which typical value is 5%. The probability, *P*, that the value higher than *L* + *g* is actually greater than the limit *L* is at least 95%, i.e., “beyond reasonable doubt” [54, 55]. The size of the guard band was *g* = *ku*, where *k* = 1.65 for the decision based on one-tailed significance test at 95%; in other terms, *g* corresponded to about 0.83 U.

We considered two possibilities, calculating the uncertainty at the legal limit (*u*_L_) or at the measured value (*u*_y_). The latter gives a larger guard band, as *u* was proportional to *y*. Moreover, the uncertainty estimated for two determinations (*m* = 2) was taken into account since the THC measurement in a sample exceeding the limit must be repeated [21].

In Table 6, we reported for each legal limit, by using the top-down uncertainty (*U* = 0.1308 THC(%) + 0.0066, for *m* = 2), the maximum THC content (*L* + *g*) beyond which non-compliance of hemp samples should be declared.

**Table 6:** Maximum THC contents beyond which hemp samples should be declared non-compliant with the various legal limits

Limit (%THC)	*L* + *g*_L_ (guard band at limit)	*L* + *g*_y_ (guard band at value)
0.20	0.23 (+15%)	0.23 (+15%)
0.30	0.34 (+13%)	0.34 (+13%)
0.50	0.56 (+12%)	0.57 (+14%)
0.60	0.67 (+12%)	0.68 (+13%)
1.00	1.11 (+11%)	1.13 (+13%)

The differences between the two approaches resulted minimal and measurable only for the higher limits. It is worth to note that at the 0.2% limit, the band guard coincides with the tolerance (0.03%) applied by the EU method, supporting the choice of the top-down uncertainty.

## Conclusion

The precision data and measurement uncertainty of the EU method for THC determination in hemp, investigated in the range 0.2–1.0%, showed a linear dependence with THC content.

We evaluated measurement uncertainty, essential to define decision rules for compliance assessment, by both bottom-up and top-down approaches and the latter resulted more suitable for the purpose.

We proposed decision rules for each THC legal limit, which resulted consistent with the strategy adopted by the EU method to determine the tolerance for the 0.2% limit: hemp samples should be declared as non-compliant when THC content, as mean result on a duplicate analysis, exceeds the set limit by 11–15%, depending on THC limit.

Here, we also want to highlight some issues concerning practical and crucial aspects that may arise during hemp analysis.

The sample size recommended by the EU method to be representative of the hemp field is quite large, comprising parts of 50 or 200 plants per field and this may cause difficulty to most analytical laboratories, as samples must be dried within 48 h.

Hemp compliance assessment takes into account the average THC value determined on the representative sample of the field. This means, however, that some single inflorescence, marketed individually, might exceed the legal limit and become a legal question for the owner or the retailer despite it comes from a production assessed as compliant.

By applying the EU method, as any other GC method without derivatization procedures, the evaluation of THCA recovery on own system is advisable, as a possible loss during its thermal conversion into THC may occur.

Currently, only Δ^9^-THC is the parameter evaluated to allow hemp cultivation. However, recently, new phytocannabinoids were discovered, the Δ9-tetrahydrocannabutol (Δ^9^-THCB) [56] and the Δ9-tetrahydrocannabiphorol (Δ^9^-THCP) [57]. The former showed a comparable activity to that of Δ^9^-THC, while the latter resulted 33 times more active. In the next future, if such high psychotropic effects will be further confirmed, it will be appropriate to include their routine determination.

Finally, it is necessary to carry out inter-laboratory studies on hemp samples with low THC content in order to estimate the actual precision data of the EU method and to suggest, in case, a more suitable official method which will take into account all the analytical issues that may affect the reliability of the results.

## Supplementary Information

ESM 1 (PDF 571 kb)
