# Supplementary material for: Delta9-THC determination by the EU official method: evaluation of measurement uncertainty and compliance assessment of hemp samples
Source: Anal Bioanal Chem. 2021 Mar 23;413(13):3399–410. doi: 10.1007/s00216-021-03283-x (PMC8105209; doi:10.1007/s00216-021-03283-x)
Supplement: Supplementary file 1 — (PDF 571 kb) [file 216_2021_3283_MOESM1_ESM.pdf]

**Delta9-THC determination by the EU official method: evaluation of measurement uncertainty and compliance assessment of hemp.**

Sgrò S.[a], Lavezzi B.[b], Caprari C.[a], Polito M.[a], D'Elia M.[c], Lago G. [d], Furlan G.[d], Ferri E.[b*], Girotti S.[b]
[a] *Chemical Laboratory of Bologna, Anti-Fraud and Controls Office – Laboratories Section, DT VII, Italian Customs and Monopolies Agency, V. le P. Pietramellara 1/2, 40121 Bologna, Italy*
[b] *Department of Pharmacy and Biotechnology, University of Bologna, Via San Donato 15, 40127 Bologna, Italy*
[c] *Scientific Police Center of Emilia-Romagna Region, Via Volto Santo 3, 40123, Bologna, Italy*
[d] *Carabinieri Scientific Investigation Group, Parco Ducale 3, Parma, Italy*

# Supplementary Information

**Bottom-up approach to uncertainty measurement evaluation.**

The GUM provides a broad indication of the possible level of uncertainty associated with the method rather than a measurement. It does not take into account either matrix-associated errors or the actual day-to-day variation seen in a laboratory.
According to the bottom-up approach reported in the GUM, in case of independent input quantities, $u_c(y)$ is given by:

$$u_c(y) = \sqrt{\sum_{i=1}^{N}\left(\frac{\partial f}{\partial x_i}\right)^2 u^2(x_i)} = \sqrt{u_A^2(y) + u_B^2(y)} \qquad \text{(S1)}$$

Where y, the % THC was calculated by the following formula:

$$THC(\%) = \frac{A_{THC}/A_{IS} - a}{b} * \frac{C_{IS} \;\; V_{sol}}{w_s} \; 100$$

*a* and *b* are respectively the intercept and the slope of the linear regression equation y=a+bx; *A* is the chromatographic peak area, *C* is the concentration in mg/ml, $V_{sol}$ is the extraction solution volume in ml and $w_s$ *is* the sample test weight in mg.

$\left(\frac{A_{THC}}{A_{IS}} = a + b\frac{C_{THC}}{C_{IS}}\right)$,

Therefore, the relative combined standard uncertainty, $\dot{u}_c(y)$, was calculated as:

$$\dot{u}_c(y) = \sqrt{\dot{u}_{rep}^2 + \dot{u}_{reg}^2 + \dot{u}_{CRM(THC)}^2 + \dot{u}_{CRM(IS)}^2 + \dot{u}_{w_s}^2 + \dot{u}_{V_{sol}}^2} \qquad (1)$$

combining the contributions due to repeatability ($u_{rep}$), (type A evaluation), to regression curve ($u_{reg}$), to THC Certified reference material ($u_{CRM(THC)}$), to IS Certified reference material ($u_{CRM(IS)}$), to sample test weight ($u_{ws}$), and to extraction solution volume ($u_{Vsol}$), all representing a type B evaluations on uncertainty.
Each input component to uncertainty budget was calculated as follow.

- **repeatability**,

$$\dot{u}_{rep} = \frac{s_r}{y\sqrt{m}} \qquad \text{(S2)}$$

where m is the number of analysis replicates and y is the THC(%) content; $s_r$, as shown, is a function of THC(%);

- **regression curve**,

$$\dot{u}_{reg} = \frac{u_{reg}}{C_{THC}/C_{IS}}, \qquad \text{(S3)}$$

Where $$u_{reg} = \frac{s_{y/x}}{b}\sqrt{\frac{1}{m} + \frac{1}{n} + \frac{(y_0 - \bar{y})^2}{b^2\sum_{i=1}^{n}(x_i - \bar{x})^2}} \qquad \text{(S4)}$$

$s_{y/x}$ is the residual standard deviation (of data points from the regression line);
m is the number of replicates on the sample,
n is the number of calibration points (multiplied by the number of replicate measurements at each point),
$y_0$ is the sample test average response (areas ratio),

$\bar{y}$ and $\bar{x}$ are the average value of $y_i$ and $x_i$ (concentrations ratio) in the calibration set, and the summation is over all data pairs.

However, we employed a modified version of this expression since the first term under the radical 1/m of the linear regression uncertainty equation is to be considered superfluous and was omitted to avoid double counting and overestimate calibration uncertainty. Indeed, as well explained by Kadis *[Kadis R. Evaluation of the measurement uncertainty: Some common mistakes with a focus on the uncertainty from linear calibration. Journal of chromatography. A. 2017.1499: 226-229. https://doi.org/10.1016/j.chroma.2017.03.078]* the component $\frac{s_{y/x}}{b\sqrt{m}}$ in this equation characterizes the estimated precision from a calibration experiment, meanwhile the observed precision (estimated from replicate determinations) is directly included in the budget of $\dot{u}_c(y)$, by the term $\dot{u}_{rep}$ that incorporates all the individual sources of variability, including that relating to calibration.

The regression uncertainties $u_{reg}$ refer, as the input data, to the THC and IS concentrations ratio, $C_{THC}/C_{IS}$, (Table S1) nevertheless the relative regression uncertainties $\dot{u}_{reg}$ are associable both to $C_{THC}$ (mg/ml) and to the corresponding %THC. In Fig. S1 is plotted the $\dot{u}_{reg}$ vs THC(%), showing a potential-type relation curve, in order to extrapolate the estimated relative regression uncertainty for the THC concentration of our interest.

**Table S1** Relative regression uncertainty associable both to $C_{THC}$ (mg/mL) and to the corresponding % THC.

| $C_{THC}$ (mg/ml) | THC (%) | $\dot{u}_{reg}$ |
|---|---|---|
| 0.02 | 0.10 | 0.0743 |
| 0.04 | 0.20 | 0.0354 |
| 0.12 | 0.60 | 0.0099 |
| 0.25 | 1.25 | 0.0047 |
| 0.50 | 2.50 | 0.0045 |

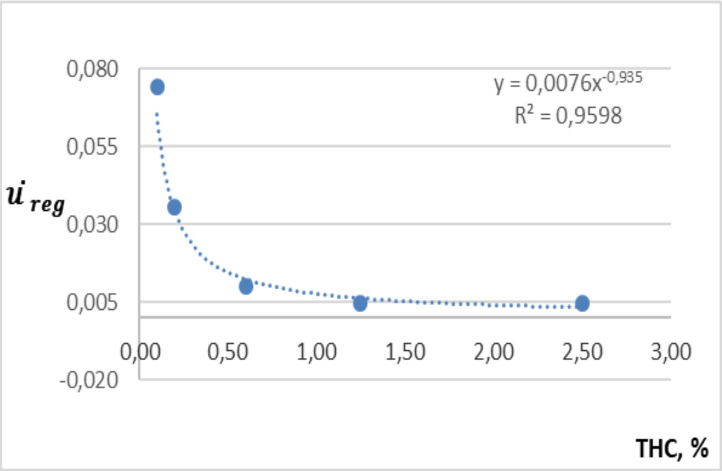


**Fig. S1** Correlation between the relative regression uncertainty and the THC content

**- THC Certified reference material**:

$$\dot{u}_{CRM(THC)} = \sqrt{\dot{u}^2_{P_{THC}} + \dot{u}^2_{\mu pip}}$$

where contribution deriving from CRM purity ($P_{THC}$) and micropipettes volume (µpip) are considered.

In turn,

$$\dot{u}_{P_{THC}} = \frac{d_{P_{THC}}}{P_{THC}\sqrt{3}},$$

where $d_P$ is the value of the confidence interval for purity provided by the manufacturer;

$$\dot{u}_{\mu pip,i} = \frac{\sqrt{\dot{u}^2_{acc,i}+\dot{u}^2_{prec,i}}}{\sqrt{3}},$$

that combines accuracy and precision of micropipette, which depend from the withdrawn volume.

The factor $\sqrt{3}$ has been introduced because the assessment of the variability range for this parameter is not based upon statistical evaluation but on an interval characterized by two extremes out of which no value is allowed and within which every value is equally probable, so called "square" probability distribution.

An average value was obtained, considering the volume range 20-500µl used for calibration points, as follow:

$$\dot{u}_{\mu pip} = \sqrt{\frac{\sum_1^i \dot{u}^2_{\mu pip,i}}{i}},$$

**- IS Certified reference material**:

$$\dot{u}_{CRM(IS)} = \sqrt{2(\dot{u}^2_{w_{IS}} + \dot{u}^2_{V_{fl}} + \dot{u}^2_{P_{IS}}) + \dot{u}^2_{\mu pip}}$$

where contribution deriving from weight ($w_{IS}$), flask volume ($V_{fl}$), purity ($P_{IS}$) and micropipette volume (µpip) are considered. IS CRM solution is used both for calibration standard solutions preparation and as sample extraction solution. Therefore, the first three terms affect twice the overall uncertainty.

In turn, $\dot{u}_{w_{IS}} = \sqrt{2}\frac{u_{bal}}{w_{IS}}$,
where $\sqrt{2}$ takes into account a double similar weighing, considering the tare, $u_{bal}$ is the standard uncertainty of the analytical balance associated to $w_{is}$, that is the IS weight;
$\dot{u}_{V_{fl}} = \frac{d_{fl}}{V_{fl}\sqrt{6}}$,
where $d_{fl}$ is the value of the confidence interval of the flask provided by the manufacturer and, in this case, a "triangular distribution" is adopted, using the factor $\sqrt{6}$.
$\dot{u}_{P_{IS}}$ and $\dot{u}_{\mu pip}$ are estimate as for THC CRM. In this case, the accuracy and precision of micropipette are those referred to 1000µl, used for calibration points preparation.

- **Sample test weight**:
$\dot{u}_{w_s} = \sqrt{2}\frac{u_{bal}}{w_s}$, considering the tare as well.

- **Extraction solution volume**:
$\dot{u}_{V_{sol}} = \frac{d_{pip}}{V_{pip}\sqrt{3}}$,
where $d_{pip}$ and $V_{pip}$ are the values of the confidence interval and the volume of the 5ml-pipette.

----

For each hypothetical legal limit in the range 0.2-1.0% of THC, the combined standard uncertainty, $u_c(y) = y\,\dot{u}_c(y)$, and the expanded uncertainty, $U(y) = k\,u_c(y)$, were calculated both for m=1 and for m=2.
Effective degrees of freedom $\nu_{eff}$ were obtained from the Welch-Satterthwaite formula considering $\dot{u}_{rip}$ and $\dot{u}_{reg}$, that is taking into account only those standard uncertainty components that have been evaluated statistically from repeated observations (for the other type B inputs, it is possible to assume infinite degrees of freedom):

$$\nu_{eff} = \frac{[\dot{u}_c(y)]^4}{\sum_{i=1}^{N}\frac{[\dot{u}(x_i)]^4}{\nu_i}}$$

Expanded uncertainty is calculated using a coverage factor of 2, as $\nu_{eff}$ >10, which gives a level of confidence of approximately 95%.
It is noteworthy, however, as the GUM (in section G 1.2) points out that in most practical measurement situations, the calculation of intervals having specified levels of confidence is at best only approximate.
In Figure S2 we represented the % contribution of the various components to the combined Standard uncertainty.

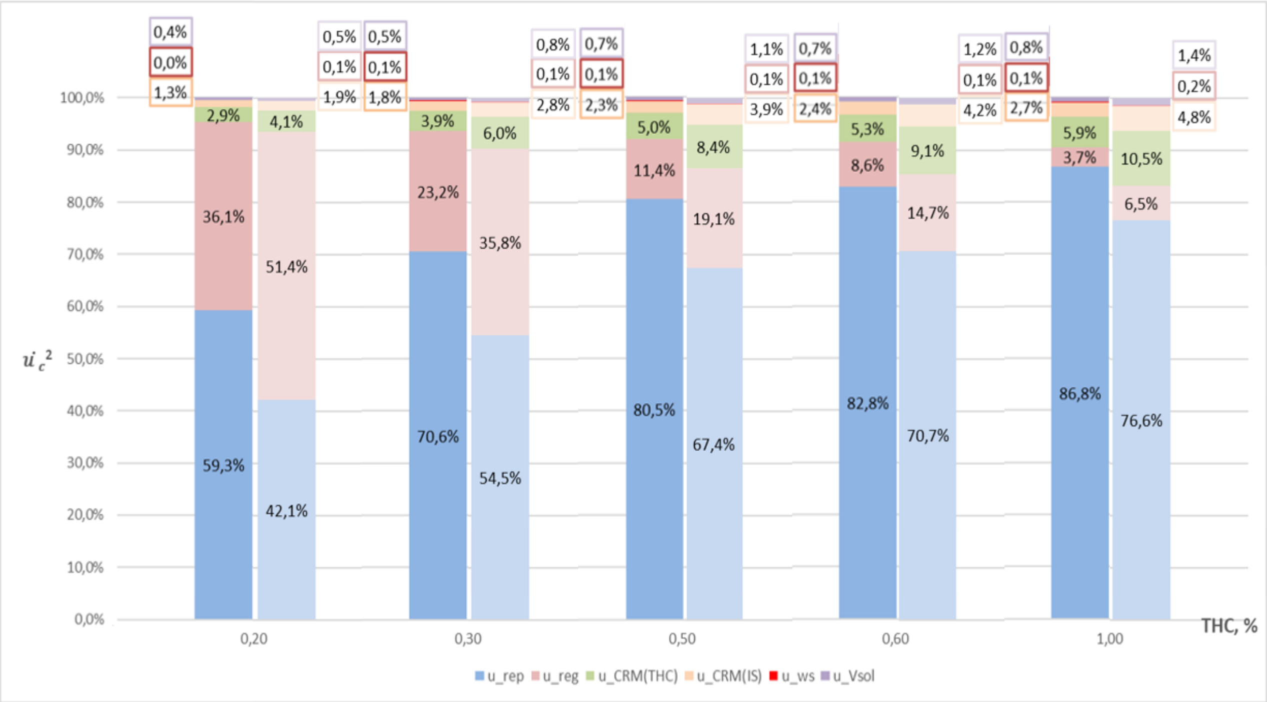


**Fig. S2** Percent contribution of each input component to combined standard uncertainty. For each THC % content: First column m=1, second column m=2.

Table S2 Comparison of uncertainty evaluations ratio values of the combined standard uncertainties estimated by both approaches, for one and two replicates analysis. For further comparison, a predicted standard uncertainty ($u_H$), based on the predicted reproducibility standard deviation ($\sigma_H$), was calculated by the Horwitz equation.

| THC (%) | $\frac{u(y)_{T\text{-}D}}{u(y)_{B\text{-}U}}$ | | $u_H$ | |
|---|---|---|---|---|
| y | m=1 | m=2 | m=1 | m=2 |
| 0.20 | 1.54 | 1.72 | 0.010 | 0.008 |
| 0.30 | 1.68 | 1.95 | 0.014 | 0.011 |
| 0.50 | 1.80 | 2.17 | 0.022 | 0.018 |
| 0.60 | 1.82 | 2.22 | 0.026 | 0.020 |
| 1.00 | 1.86 | 2.32 | 0.040 | 0.030 |

The values of $u_H$ were calculated by $u(y) = \sqrt{s_R^2 - s_r^2(1 - \frac{1}{m})}$ assuming as mentioned $s_R=\sigma_H$.
